# Symptomatic Pulmonary Hamartoma

**DOI:** 10.7759/cureus.18230

**Published:** 2021-09-23

**Authors:** Isha Shukla, Thor S Stead, Ilya Aleksandrovskiy, Vashun Rodriguez, Latha Ganti

**Affiliations:** 1 Emergency Medicine, Trinity Preparatory School, Winter Park, USA; 2 Medicine, The Warren Alpert Medical School of Brown University, Providence, USA; 3 Emergency Medicine, Ocala Regional Medical Center, Ocala, USA; 4 Department of Emergency Medicine, Lakeland Regional Health, Lakeland, USA; 5 Emergency Medicine, Envision Physician Services, Plantation, USA; 6 Emergency Medicine, University of Central Florida College of Medicine, Orlando, USA; 7 Emergency Medicine, HCA Healthcare Graduate Medical Education Consortium Emergency Medicine Residency Program of Greater Orlando, Orlando, USA

**Keywords:** lung tumor, pulmonology, emergency medicine, exertional dyspnea, pulmonary hamartoma

## Abstract

Pulmonary hamartoma is the most common benign tumor of the lungs. It is most often asymptomatic and is discovered incidentally. The condition is two to three times more common in men and is usually seen in the sixth to seventh decade of life. The authors present the case of a 44-year-old female in whom the condition was symptomatic, causing cough, shortness of breath, and fatigue. This case is unusual in that it occurred in a woman in her 40s and was symptomatic. The authors discuss the presentation, clinical features, and management of pulmonary hamartoma.

## Introduction

Pulmonary hamartoma is a term coined in 1904 by the German pathologist Eugen Albrecht that describes benign tumors occurring in the lungs. It is the most common benign pulmonary tumor among adults [1], comprising 77% of all benign lung tumors. Two large autopsy-based studies reported an incidence of 0.025% and 0.032% [2]. The condition is more common among men than women and elderly populations [3-5]. Because the majority of patients with pulmonary hamartoma are asymptomatic [5], it is typically discovered incidentally through a computerized tomography (CT) scan and/or chest radiograph. Pulmonary hamartoma often presents as a well-defined, coin-shaped mass in the periphery of the lungs [6]. The calcification of the tumor has a comma-shaped or popcorn appearance [7]. It is often accompanied by other tumors and lesions such as those seen in Cowden syndrome [8]. Patients with pulmonary hamartomas have 6.3 times the risk of lung cancer compared to the general population after adjusting for age, sex, and ethnicity [9]. The primary treatment option is surgical resection followed by regular follow-ups to detect the potential recurrence of tumors [10]. If left untreated, the tumor can continue to grow slowly and cause symptomatic patients to experience worsening symptoms [1].

## Case presentation

A 44-year-old female presented to the emergency department complaining of fatigue. She had an episode of chest pain approximately 15 hours before the presentation that lasted several minutes and subsided. She also had a right-sided flank and upper torso discomfort. She exercised regularly and reported that although it could have been a muscle pull, it felt different. The day prior she had experienced one episode of feeling slightly short of breath. Both the pain and shortness of breath had resolved, and she had been asymptomatic for the last 15 hours. The symptom that concerned her the most was extreme tiredness. She explained that for the last three days, she had been feeling incredibly tired despite being very active with a full-time job during the day and raising a family. She was concerned because she was wondering if her tiredness was caused by her thyroid. The patient had a thyroidectomy at age 18 for a nodule and had been on thyroid supplementation ever since. She requested to get her thyroid level checked. The patient had no risk factors for coronary artery disease, nor did she have a family history of coronary artery disease or stroke. In addition to the thyroid medication, she did not take any other medications. The patient had a normal body mass index and was a nonsmoker.

Her vital signs were temperature of 97.8°F, blood pressure 140/63 mmHg, pulse rate 54 beats per minute, respiration rate 15 breaths per minute, and oxygen saturation of 100%. The patient’s laboratory results were unremarkable (Table 1).

**Table 1:** Patient’s laboratory values.

Lab test	Result	Reference value
Sodium	138	135–145 mmol/L
Potassium	4.0	3.5–5.3 mmol/L
Chloride	106	98–107 mmol/L
Carbon dioxide	26	21–32 mmol/
Blood urea nitrogen	18	7–18 mg/dL
Creatinine	0.8	0.6–1.3 mg/dL
Glucose	113	74–106 mg/dL
Calcium	9.0	8.4–10.2 mg/dL
Troponin I	<0.01	0.02–0.05 ng/mL
Thyroid-stimulating hormone	0.47	0.36–3.74
D-Dimer	306	0–500 ng/mL FEU
White blood cell count	6.9	4.1–9.3 K/mm^3^
Red blood cell count	4.25	3.28–5.50 M/mm^3^
Hemoglobin	13.4	12.1–15.1 g/dL
Hematocrit	4.04	12.1–15.1 g/dL
Platelet count	172	150–450 K/mm^3^

**Figure 1:**
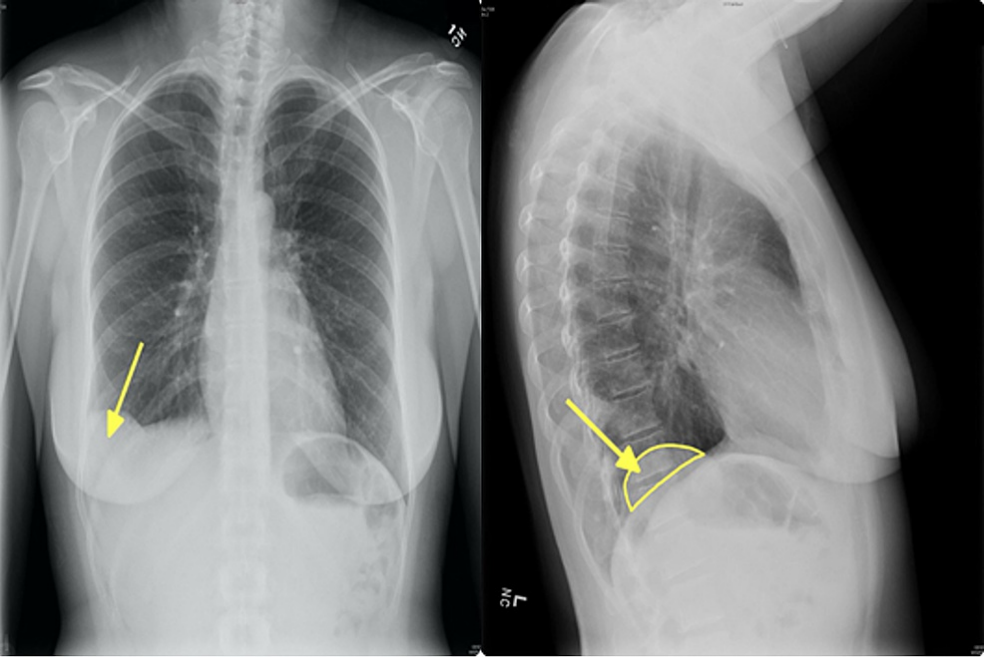
Chest radiograph anteroposterior and lateral views demonstrating a mass in the right costophrenic angle (arrow) and diaphragmatic eventration on the left (semicircle).

Urinalysis revealed moderate hematuria. A chest radiograph demonstrated a contour abnormality at the right hemidiaphragm suggesting lateral eventration of the diaphragm and a pulmonary mass on the right (Figure 1).

**Figure 2:**
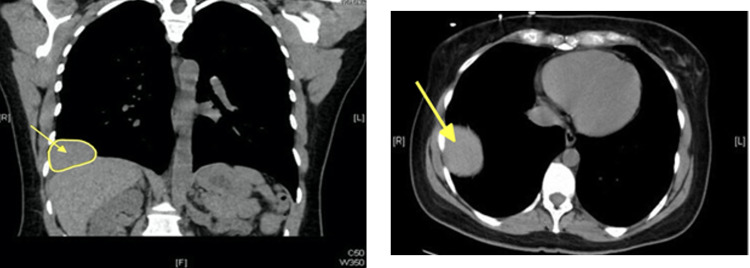
Chest computed tomography axial and coronal views demonstrating right-sided solitary pulmonary mass.

Chest CT revealed a large mass in the right lateral costophrenic recess. The imaging features were consistent with a benign pulmonary tumor such as hamartoma, given the suggestion of internal fatty features and lack of erosion or compromise of the adjacent chest wall (Figure 2).

## Discussion

This case highlights the elusiveness of pulmonary hamartoma and the importance of CT scans and chest radiographs in confirming the diagnosis of pulmonary hamartoma. This case is atypical because the patient was a 44-year-old female and was not a part of either of the risk populations for pulmonary hamartoma (male and elderly) [2]. While pulmonary hamartoma typically does not cause symptoms, the patient experienced fatigue and dyspnea. Moreover, her symptoms were common pulmonary symptoms that could have been attributed to cardiovascular or pulmonary conditions such as heart failure or chronic obstructive pulmonary disease or simply stress, and thus potentially overlooked. Her CT scans and radiographs revealed a classic presentation of pulmonary hamartoma: a well-defined, round mass. She also had a diaphragmatic eventration that was most likely incidental as there is no known relationship between pulmonary hamartoma and diaphragmatic evisceration.

Pulmonary hamartoma can be treated through surgical resection such as tumor enucleation, wedge resection, and lobectomy for more serious cases [10]. A retrospective study of 226 patients with pulmonary hamartoma revealed that only one patient died after surgical resection, indicating that it is a low-risk procedure. When surgery is not viable, the tumor can be left untreated and typically has a good prognosis [11]. However, in rare cases, the tumor can show rapid growth [12], suggesting that it may be malignant. A review of multiple studies has concluded that resection should be reserved for symptomatic patients to avoid postoperative mortality or the development of malignant lesions in asymptomatic patients. Nevertheless, observation of the tumor is advised to detect the occurrence of malignant tumors as the possibility of developing lung cancer is six times higher in patients with pulmonary hamartoma [9].

## Conclusions

Pulmonary hamartoma is a benign pulmonary tumor that can sometimes result in pain or dyspnea if it is sufficiently large. This case highlights such an occurrence and is unusual because the patient did not have any known risk factors for a pulmonary tumor. Although pulmonary hamartoma is more commonly noted in men and in the sixth and seventh decades of life, it can and does occur in younger people and in women, as this case highlights. Making the diagnosis is important for both surveillance and the decision to intervene.
